# Nephrogenic adenoma of the urinary bladder. Clinical features, long-term follow-up and recurrence predictors

**DOI:** 10.1080/20905998.2024.2402585

**Published:** 2024-09-11

**Authors:** Mohamed H Zahran, Mohamed Shehata, Shery Khater, Hosam Nabeeh, Mohamed Ali Badawy, Ahmed Mosbah, Hassan Abol-Enein

**Affiliations:** a Urology Department, Urology and Nephrology Center, Mansoura University, Mansoura, Egypt; b Pathology Department, Urology and Nephrology Center, Mansoura University, Mansoura, Egypt; c Urology Department, Kafr Elsheikh University, Kafr Elsheikh, Egypt; d Radiology Department, Urology and Nephrology Center, Mansoura University, Mansoura, Egypt

**Keywords:** Bladder cancer, nephrogenic adenoma, cystoscopy, tumour resection, recurrence

## Abstract

**Objectives:**

To review the clinical features of nephrogenic adenoma (NA) of the urinary bladder and assess the long-term recurrence rates.

**Material and methods:**

A retrospective analysis of all diagnosed NA of the urinary bladder between 2000 and 2023 was done. All patients’ demographics, symptoms, history of previous bladder surgeries, macroscopic and microscopic features of NA and coincidental pathology were reviewed. The long-term recurrence rate including malignant recurrence was assessed. Univariate and multivariate analysis of predictor of recurrence was performed.

**Results:**

We identified 24 patients diagnosed with NA with a mean (SD, range) age of 56 (16, 15–73) years. Eighteen (75%) patients had previous history of bladder surgeries. Macroscopic appearance on cystoscopy looked polypoidal, flat velvety, papillary and nodular in eight, seven, five, and four patients, respectively. At a median (range) follow-up of 35 (18–145) months, 15 (63%) patients developed one recurrence including NA, non-specific cystitis and urothelial carcinoma (UC) in nine, three and three patients, respectively. Of them, five had a second recurrence, and one patient had a third recurrence. Patients with malignant recurrence had previous history of UC and two of them required radical cystectomy and urinary diversion. Previous TURBT was the only independent predictor for recurrence [OR (95% CI) = 28 (2–37), *p* = 0.01].

**Conclusion:**

Nephrogenic adenoma is a benign disease that mimics malignant lesion clinically. It is associated with high recurrence rate. Previous resection of bladder lesion is the independent predictor of the recurrence.

## Introduction

Nephrogenic adenoma (NA) is an uncommon benign lesion of the urinary tract. It is strongly associated with chronic irritation or inflammation of the urinary tract from previous bladder surgery, resection of bladder lesion, bladder augmentation, chronic catheterization, chronic urinary tract infection, urolithiasis and intra-vesical therapy. Weather it is a metaplasia of the urothelium in response to chronic irritation or of embryonic origin from mesonephric tissue, it did not affect the inherent nature of the disease [1].

It is a rare pathological finding observed in association with hematuria or irritative lower urinary tract symptoms (LUTS). Although its diagnosis is straightforward in most cases, it mimics a variety of malignant lesions clinically and pathologically [2]. Certain immunohistochemical stains like PAX-8 might be indicated for proper diagnosis [3].

Symptoms usually regress after endoscopic resection, with high possibility of recurrence [4]. Some reports of malignant association raised the suspicious of being premalignant condition [5]. However, the available data in the literature do not support this elucidation [2]. Herein, we reviewed our database of all diagnosed cases of NA of the urinary bladder and evaluated the long-term outcome in terms of recurrence and malignant transformation.

## Material and methods

After obtaining institutional review board approval (R.24.03.2530), we revised the histopathological reports of all patients who underwent transurethral resection for bladder tumour (TURBT) between January 2000 and June 2023. Patients with pathologically proven diagnosis of bladder NA were eligible for evaluation. Archived specimen for diagnosed cases were reviewed by an expert uropathologist. Furthermore, retrospective review of all dedicated electronic database including clinical, pathological and radiological characteristics was performed.

### Intervention

In all cases, TURBT was performed for all visible lesions under spinal anesthesia using a 26 Fr resectoscope. Complete resection was done for all visible lesions deep to the underlying muscles. The bladder was then drained using 20 Fr Foley’s catheter and kept for 48 h. In case of NA, follow-up cystoscopy was recommended after 3 months and then after 1 year. Any suspicious lesion at follow-up cystoscopy is an indication for admission for further endoscopic resection.

### Data collection

All patients’ demographic criteria were retrieved including age at diagnosis, gender, medical comorbidities, BMI, presenting symptoms and history of previous TURBT, bladder surgeries, intravesical BCG, recurrent UTI, vesical stones and antecedent bladder pathology. Cystoscopy findings including the lesion site, macroscopic features and relation to the site of the previous lesions were fully reviewed. Also, microscopic features and concomitant pathology were re-assessed.

The onset of recurrence and the histopathology of recurrent lesion were reviewed at last follow-up. Univariate analysis of all demographic and clinical criteria associated with disease recurrence was done.

### Statistical analysis

Continuous variables were expressed as mean ± SD or median (range) according to the pattern of distribution, where categorical variables were expressed as number (percentage). Univariate analysis of factors affecting recurrence was done using Chi-square test and independent sample t-test. Multivariate analysis was done using binary logistic regression analysis. All statistical tests were done using IBM SPSS statistics version 21, with two-sided tests, and a *p* value of less than 0.05 was considered statistically significant.

## Results

### Clinical features

In the determined period, 4352 patients had undergone TURBT for bladder lesions. We identified 24 (0.55%) cases of NA of the urinary bladder in 21 males and 3 females. The mean (SD, range) age was 56 (16, 15–73) years. Irritative LUTS, hematuria and both were the presenting symptoms in 13 (54%), 4 (17%) and 7 (29%) cases, respectively. History of previous 18 TURBTs was identified in 12 (50%) cases. The number of previous TURBT was once, twice and thrice in seven, four and one cases, respectively. The histopathology was high-grade urothelial carcinoma (UC), low-grade UC and chronic non-specific cystitis (CNSC) in 11, 6 and 1 case, respectively. One patient had undergone right nephroureterectomy and bladder cuff excision for upper tract UC, followed by two TURBTs for low-grade papillary UC. Five patients had a history of prostatic surgeries (four; open prostatectomy and one; TURP), and one patient had augmentation ileocystoplasty. Nephrogenic adenoma was diagnosed after a median (range) of 18 (3-48) months after previous bladder procedure. Patients’ demographic criteria are illustrated in Table 1.

**Table 1.** Patients’ demographic criteria and pathological features of nephrogenic adenoma.

	No (%)
**Age**. *Years. Mean ± SD*	56 ±16
*2^nd^ decade*	1 (4.2%)
*3^rd^ decade*	6 (25%)
*4^th^ decade*	4 (16.7%)
*5^th^ decade*	2 (8.3%)
*6^th^ decade*	7 (29.2%)
*7^th^ decade*	4 (16.7%)
**Gender**	
*Male*	21 (88%)
*Female*	3 (12%)
**BMI**	
*Mean ± SD*	29±4.6
**Symptoms**	
*Irritative LUTS*	13 (54%)
*Hematuria*	4 (17%)
*Both*	7 (29%)
**Medical co-morbidities**:	
*Diabetes mellitus*	7 (29%)
*Hypertension*	10 (41%)
**Smoking**	
Active s*moker*	5(20.8%)
*Ex-smoker*	3 (12.5%)
*Non-smoker*	16 (66.7%)
**Previous surgeries:**	
*TURBT*	12 (50%)
*Augmentation ileocystoplasty*	1 (5%)
*Bilateral ureterovesical anastomosis*	1 (5%)
*TURP*	1 (5%)
*Trans-vesical open prostatectomy*	4 (19%)
**Antecedent pathology:**	
*Recurrent UTI*	6 (29%)
*Vesical Stone*	4 (19%)
*Urothelial carcinoma*	12 (50%)
*CNSC*	1 (5%)
**Macroscopic features**	
*Polypoid*	8(33%)
*Papillary*	5 (21%)
*Flat velvety*	7(29%)
*Nodular*	4 (17%)
**Microscopic features**: *Tubular*	7 (29%)
*Papillary*	1 (4%)
*Flat lesion lined with cuboidal cells* *Mixed papillary and tubular*	6 (25%) 7 (29%)
*Mixed flat and tubular*	3 (13%)
**Coincident pathology:**	
*Inflammation*	16 (67%)
*Proliferative changes*	3 (13%)
*Eosinophilic cystitis*	2 (8%)
*Hyperplasia*	2 (8%)
*Squamous metaplasia*	1 (4%)

TURBT: transurethral resection of bladder tumor, TURP: transurethral resection of prostate, CNSC: chronic non-specific cystitis.

### Morphological features

**Figure 1.**
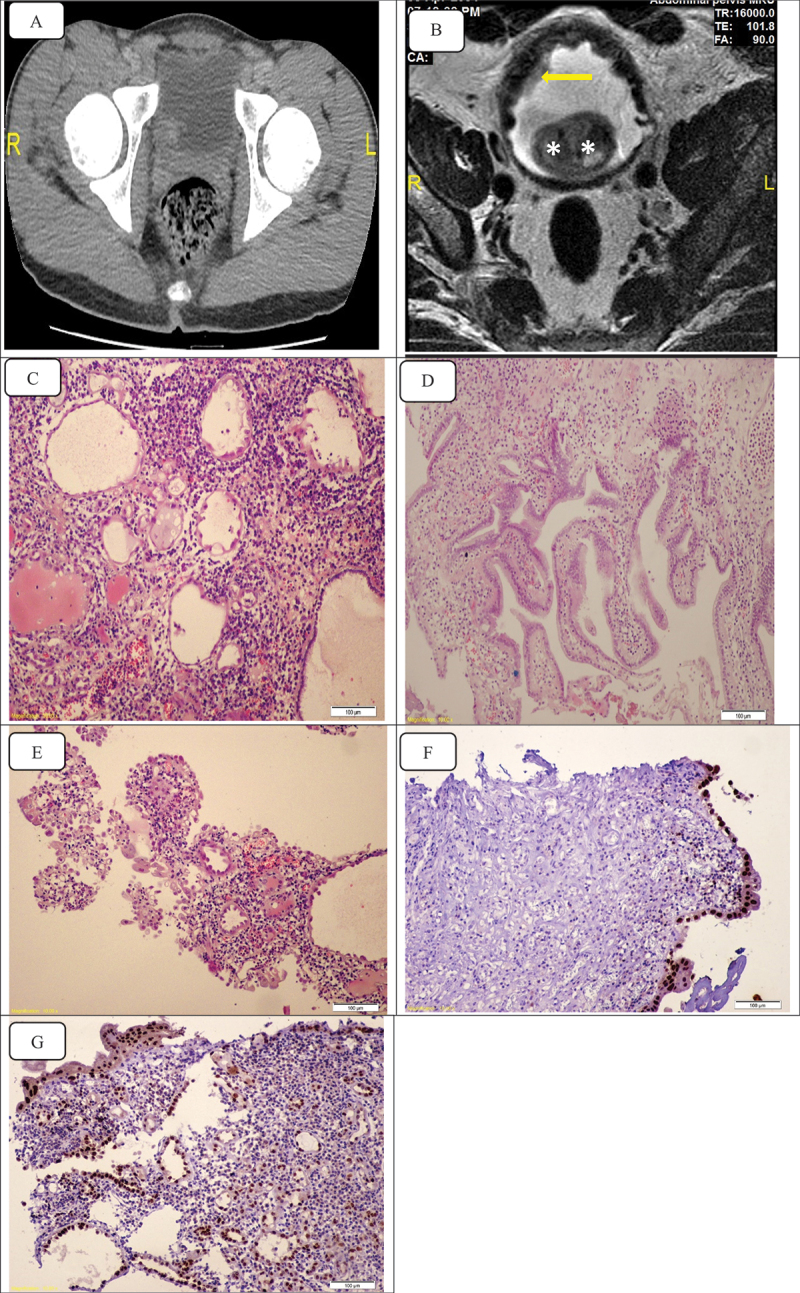
A: contrast-enhanced CT scan shows right posterolateral wall intravesical enhancing soft tissue mass encroaching upon the right ureteric orifice. B: axial T2WI pelvic MRI shows diffuse thickened serrated bladder wall with multiple small diverticula, a right lateral wall polypoidal mucosal growth (arrow) and intravesical enlarged median lobe of the prostate is seen posteriorly (asterisks). Hematoxylin and eosin (H&E) stain of a nephrogenic adenoma composed of (C) tubular structure lined with single layer of cuboidal cells (lacking cellular atypia) surrounded by distinct basement membrane with edematous mildly inflamed lamina propria. D: papillary structure, E: mixed papillary and tubular structures, F: flat lesion stained with PAX-8, G: flat and tubular lesion stained with PAX-8.

Macroscopically, the lesion was polypoidal (eight cases, 33%), flat velvety (seven cases, 29%), papillary (five case, 21%) and nodular (four cases, 17%). The lesions were located at the posterior wall (12 cases, 50%), the lateral wall (7 cases, 29%), the bladder neck (2 cases, 8%), multicentric (2 cases, 8%) and in bladder diverticulum (1 case). It was located at the same site of the previously resected bladder lesions in nine cases (75%) and at other sites in the other three cases. All cases were treated by TURBT. Microscopically, the lesions were tubular, papillary, flat lesion lined with cuboidal cells, mixed papillary and tubular and mixed flat and tubular in seven (29%), one (4%), six (25%), seven (29%) and three (13%) cases, respectively. No atypia, mitosis or invasion of the basement membrane was identified in all lesions. All were stained positive with PAX8 immunohistochemical stain. Coincidental lesions with NA were inflammation, proliferative changes (cystitis cystica and glandularis), eosinophilic cystitis, hyperplasia and squamous metaplasia in 16, 3, 2, 2 and 1 case, respectively (Figure 1).

### Recurrence

**Figure 2.**
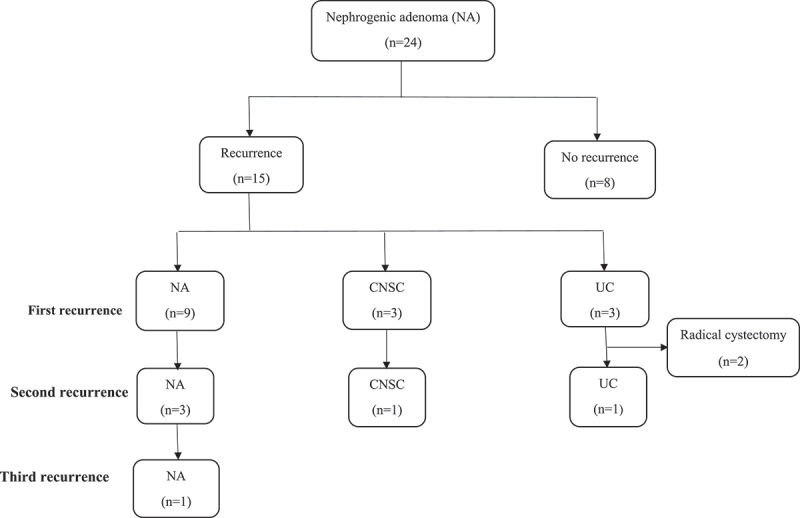
Illustration of the long-term outcome of nephrogenic adenoma (NA: nephrogenic adenoma, CNSC: chronic non-specific cystitis, UC: urothelial carcinoma).

At a median (range) follow-up of 35 (18–145) months, 15 (63%) patients developed recurrence (Figure 2). The first recurrence was reported after a median (range) of 15 (3-73) months. Recurrence was identified at the same site of previous resection in 11 patients and at a different site in 4 patients. The pathology of first recurrence was NA, CNSC and UC in nine, three and three cases, respectively.

Second recurrence had been reported in five patients after a median (range) of 3.5 (3-12) months. The pathology was similar to the first recurrence in all cases (three with nephrogenic adenoma, one with CNSC and one with high-grade UC). In one patient, three recurrences with NA had been reported.

Patients with malignant recurrence had a previous history of UC. Two of them were at the same site of the nephrogenic adenoma and the previous resected high-grade UC. Both of them required radical cystectomy (RC) for T2a NO high-grade UC with glandular differentiation. In the first case, RC and ileal conduit was performed 23 months after resection of the previous NA. The second patient underwent RC and continent cutaneous diversion 46 months after resection of the NA. The patient is disease free after 35 months of follow-up. The third case had high-grade UC recurrence after 4 months at the previous site of NA but at a different site from the previously resected low-grade UC. He received intra-vesical BCG and developed another recurrence 12 months later. It was treated by TURBT, and the diagnosis was low-grade UC. After 95 months of follow-up, he is alive and disease free.

In a univariate analysis, previous TURBT and intravesical BCG were associated with a statistically significant high recurrence rate (p = 0.003 and 0.01, respectively). In a multivariate analysis, previous TURBT was the independent predictor of recurrence with OR (95% CI) of 28 (2–37) and *p* value = 0.01 (Table 2).

**Table 2.** Univariate analysis of factors predicting recurrence.

	Recurrence	No recurrence	P value
**Age**. *Years#* *Mean ± SD*	52.6±14	55.7±19	0.6
**BMI**. *Mean ± SD#*	29.3±5	28±4	0.6
**Diabetes Mellitus**. *No (%)*	3 (43%)	4 (57%)	0.2
**Hypertension**. *No (%)*	7 (70%)	3 (30%)	0.5
**Recurrent UTI**. *No (%)*	3 (50%)	3 (50%)	0.4
**History of vesical stone**. *No (%)*	3 (75%)	1 (25%)	0.5
**Previous bladder surgery**. *No (%)*	13 (72%)	5 (28%)	0.08
**Smoking**. *No (%)*	5 (62.5%)	3 (37.5%)	0.6
**Previous TURBT**. *No (%)*	11 (92%)	1 (8%)	**0.003**
**Number of TURBT**. *No (%)*			0.3
*Single*	7 (100%)	0	
*Multiple*	4 (80%)	1(20%)	
**Previous intravesical BCG**. *No (%)*	9 (90%)	1 (10%)	**0.01**
**Macroscopic features**. *No (%)*			0.3
*Flat velvety*	6 (86%)	1 (14%)	
*Polypoid*	5 (63%)	3 (37%)	
*Papillary*	2 (40%)	3 (60%)	
*Nodular*	2(50%)	2 (50%)	
**Lesion site**. *No (%)*			0.8
*Posterior wall*	9 (69%)	4 (31%)	
*Lateral wall*	4(57%)	3 (43%)	
*Others*	2 (50%)	2 (50%)	
**Microscopic features**. *No (%)*			0.1
*Flat lesion lined with cuboidal cells*	6 (100%)	0	
*Tubular*	3 (43%)	4 (57%)	
*Papillary*	0	1 (100%)	
*Mixed papillary and tubular*	4 (57%)	3 (43%)	
*Mixed flat and tubular*	2 (67%)	1 (33%)	
**Coincident pathology**. *No (%)*			0.1
*Inflammation*	12 (75%)	4 (25%)	
*Proliferative changes*	2 (67%)	1 (33%)	
*Eosinophilic cystitis*	1 (50%)	1 (50%)	
*Hyperplasia*	0	2 (100%)	
*Squamous metaplasia*	0	1 (100%)	

#Independent sample t-test; all other comparisons were done using chi-square test.

## Discussion

Since the first description of NA by Davis in 1949, few hundreds of cases were reported in the literature. Most were case reports or small case series from single centers. It was first described as hamartoma of the urinary bladder. Later on, with electron microscopic-proved similarity to the distal renal tubules, it came to be named NA [4]. It was initially described that it developed as a result of metaplasia of the urothelium in response to chronic bladder irritation. Most of the reported cases had history of bladder surgeries, recurrent UTI, urolithiasis or urethral catheterization [3,6]. Fluorescence in situ hybridization analysis of NA tissue in 29 renal transplant patients has changed the previous theory. It proved that NA developed from proliferation of exfoliated and implanted renal tubular cells in the urothelium [7].

Nephrogenic adenoma is usually detected in the fourth decade of life [8]. Yet, the range of reported age at diagnosis varied between 3 and 96 years [3,9]. Few cases were reported during childhood after bladder surgeries for exstrophy and ureteroneocystostomy [3,6]. Similarly, the range of age among our cases was (15–71) year. We diagnosed NA in adolescent aged 15 years with a previous history of bilateral ureteroneocystostomy. Males were more affected with 2–3.6-fold risk than females [6,9]. Herein, 88% of diagnosed cases were in men.

There has been a strong association between NA and chronic bladder irritation and inflammation. Previous recurrent UTI, bladder calculi, chronic catheterization, intra-vesical instillation of mitomycin or BCG, exstrophy, bladder augmentation, endoscopic resection of bladder lesions were reported as risk factors for NA [3,8,10]. Herein, all included patients had a previous history of at least one of these risk factors. It has been stated that the incidence increases among renal transplant patients [6,7]. Yet, none of our cases had a history of renal transplantation. Following bladder augmentation, NA has been reported in two studies [2,11]. In one patient with previous augmentation ileocystoplasty, a papillary NA was detected in the trigone after 27 months. It recurred 14 months later and was managed successfully with complete TURBT.

Antecedent UC was stated as the most frequent clinical lesion among patients with NA [9]. It has been reported in 29–86% of NA cases [2,3,12]. Tse et al. reported that 6 out of 22 cases were associated with UC of the bladder; four of them received intra-vesical therapy [13]. In another report, 26.7% of NA patients had undergone resection of UC and received either BCG or mitomycin C [6]. Half of our cases had a previous history of TURBT of UC. Ten (42%) patients received intra-vesical BCG. Coincidental UC was reported in previous four studies in 3–42% of NA cases [2,3,6,9]. Herein, none of our cases had coexisting UC.

NA patients typically present with hematuria or LUTS, such as dysuria, urinary frequency or even incontinence [3,6]. Some cases are asymptomatic and are discovered incidentally [13]. All our cases were symptomatic with either LUTS, hematuria or both. Cystoscopically, patients can have solitary or multiple lesions [8,13], which are often polypoid [8,12,13] or papillary [2,14], but can also be flat erythematous or concealed by other urothelial lesions [8,12]. Besides polypoid and papillary lesion, we identified flat and nodular lesion in 46% of cases. There was no specific site predilection. It was detected to affect any part of the bladder wall. Yet, there was a strong correlation with the previous resection site. Similarly, Tse et al. found that four out of six patients with previous UC had NA lesions directly over or close to the site of previous fulguration [13].

Transurethral resection of NA provides a definite diagnosis and relief of symptoms in most of the cases [2]. Some larger tumors may require partial cystectomy [2,8,12]. Intra-vesical instillation of hyaluronic acid was tried following resection of the lesion in an assumption of providing a protective effect on the glycosaminoglycan layer and delays or prevents its recurrence [15]. However, delayed recurrence (after 4 years) has been reported despite maintenance on intra-vesical hyaluronic acid [16]. In another child, after multiple surgical resections for recurrent NA, it resolved with long-term treatment with ibuprofen and trimethoprim and sulfamethoxazole [17]. However, maintenance on long-term antibiotic and/or anti-inflammatory was not proved to affect the natural history of the disease.

Recurrence is an inherent nature of NA. The incidence of recurrence varied between 3.8% and 75% of cases [2,3,6,8,12–14]. Zougkas et al. reported that three out of four cases had 1–7 recurrences [4]. Peeker et al. recorded that 29% had one or more recurrence [12]. We recorded that 63% developed recurrence. The higher recurrence rate among our patients may be explained by the longer follow-up and the strict follow-up cystoscopy protocol. Given the high likelihood of recurrence, it would be prudent to follow-up patients with cystoscopy. The exact timeline for follow-up cystoscopy is not available yet. Based on the previous reports with documented higher recurrence rate within the first year of follow-up, Yi et al. recommended a follow-up cystoscopy within 6–12 months from the initial diagnosis and then with symptom recurrence [6]. Yet, we noticed a late recurrence even after 70 months of follow-up, which necessitates longer follow-up regimen. It is worth mentioning that patients with delayed recurrence (>24 month) had a previous history of TURBT for UC. We discovered most of the recurrent lesions at the site of the previous resection. Similarly, Hartmann et al. reported multiple recurrent NA at the same site of previous resection [18]. Also, NA in bladder diverticulum has been relapsed three times at the same site within 24 months [19]. We diagnosed NA case in bladder diverticulum, which was completely resected with no evidence of recurrence. We identified that previous TURBT was directly correlated with recurrence. This may be because of the effect of the repeated surgical trauma with its associated inflammatory reaction.

The concept of transformation of NA into malignant lesion is not widely accepted. Many authors reported non-malignant transformation on long-term follow-up despite the high recurrence rate [6,13,14]. Others documented malignant transformation of NA into clear cell adenocarcinoma [18,20,21]. These observations have been explained by molecular similarity of the origin of both NA and clear cell adenocarcinoma of the lower urinary tract. This was based on the expression of a cell lineage restricted transcription factor PAX8 in both lesions and absence of its expression in either normal urothelium or other urological malignancy including prostatic adenocarcinoma [22]. Moreover, comparative genomic hybridization analysis revealed identical changes in both the clear cell adenocarcinoma and NA and additional genomic changes in chromosomes 1, 4, 8 and 9. This supported the clonal evolution of NA to clear cell adenocarcinoma [18]. In the current study, three patients developed malignant recurrence at the same site of previous NA. Two required radical cystectomies after the first recurrence because of muscle-invasive high-grade UC, and the third one had two recurrences of NMIBC and received intra-vesical BCG with no other relapses after 8 years of follow-up. It should be noted that all these patients had a previous history of resected UC. This may preclude the potential malignant transformation of NA. Yet, Zougkas et al. reported development of GII UC 3 years after NA at the same site without a prior history of UC [4].

This study has some limitations. It is a case series study that retrospectively evaluated a limited number of cases. This cannot be overcome because of the natural rarity of the disease. No genetic testing of the lesions has been performed to correlate it to the previous UC or to predict the recurrence potentials and the precancerous nature of the disease. This should be evaluated in future studies to clarify the natural history of this disease.

## Conclusion

Nephrogenic adenoma is a rare benign disease of the urinary bladder associated with bladder irritation and inflammation. It mimics the malignant lesion clinically and pathologically with reported high recurrence rate. Malignant recurrence is not uncommon, yet the exact relation to the NA is still a matter of debate. Proper management and close follow-up are mandatory to detect early recurrence, especially in those with previous history of resected UC.
